# Toll-Like Receptor 4 Activation in Cancer Progression and Therapy

**DOI:** 10.1155/2011/609579

**Published:** 2011-11-03

**Authors:** Alja Oblak, Roman Jerala

**Affiliations:** ^1^Department of Biotechnology, National Institute of Chemistry, 1000 Ljubljana, Slovenia; ^2^EN-FIST Centre of Excellence, 1000 Ljubljana, Slovenia; ^3^Faculty of Chemistry and Chemical Technology, University of Ljubljana, 1000 Ljubljana, Slovenia

## Abstract

Cancer immunotherapy has been the focus of intense research since the late 19th century when Coley observed that bacterial components can contribute to cancer regression by eliciting an antitumor immune response. Successful activation and maturation of tumor-specific immune cells is now known to be mediated by bacterial endotoxin, which activates Toll-like receptor 4 (TLR4). TLR4 is expressed on a variety of immune as well as tumor cells, but its activation can have opposing effects. While TLR4 activation can promote antitumor immunity, it can also result in increased tumor growth and immunosuppression. Nevertheless, TLR4 engagement by endotoxin as well as by endogenous ligands represents notable contribution to the outcome of different cancer treatments, such as radiation or chemotherapy. Further research of the role and mechanisms of TLR4 activation in cancer may provide novel antitumor vaccine adjuvants as well as TLR4 inhibitors that could prevent inflammation-induced carcinogenesis.

## 1. Introduction

Immune system plays a crucial role not only in defense against microbial infection but also in control and surveillance of malignant neoplasms. Immune cells scan tissues with the objective to remove newlyformed malignant cells before they turn into fully formed tumors. Malignant cells developed intricate mechanisms that enable them to inhibit immune cells through secretion of specific cytokines that create an immunosuppressive environment [1]. Tumors can even directly kill tumor-infiltrating lymphocytes, which are CD95 sensitive, by expressing the CD95L (Fas ligand) [2].

Innate immunity is the first line of defense against microbial infection. Innate immune cells recognize the intruding pathogen and trigger appropriate immune response with the help of Toll-like receptors (TLRs), arguably the most important vertebrate innate immune receptors. TLRs recognize different molecules of microbial origin, called pathogen-associated molecular patterns. TLRs are located on the cell surface (TLR1, 2, 4, 5, 6) or in the endosomal compartments (TLR3, 7, 8, 9), where they safeguard the organism against infection. After recognition of their respective ligands, TLRs dimerize and trigger a cytoplasmic signaling pathway that leads to activation of several nuclear factors (e.g., NF*κ*B, IRF) responsible for transcription of immune genes [3].

Toll-like receptor signaling in immune cells is critical for regulation of innate and adaptive immune responses, such as DC maturation and antigen presentation as well as CD8+ T-cell cytotoxicity, all of which are important factors in antitumor immunity [4]. On the other hand, TLR stimulation can also result in enhanced regulatory T-cell proliferation and suppressor function favoring tumor development [5–7]. TLR expression is not limited to immune cells, and indeed many tumor cells have been found to express TLRs, signaling through which can promote tumor growth and immune evasion [8, 9]. On the other hand, TLR signaling in tumor cells was also shown to reduce the proliferative capacity of tumor cells [10]. We will focus on reports concerning TLR4 signaling and its involvement in cancer development and progression as well as the therapeutic benefit that could come from TLR4 stimulation.

## 2. Toll-Like Receptor 4 in Health and Disease

**Figure 1:**
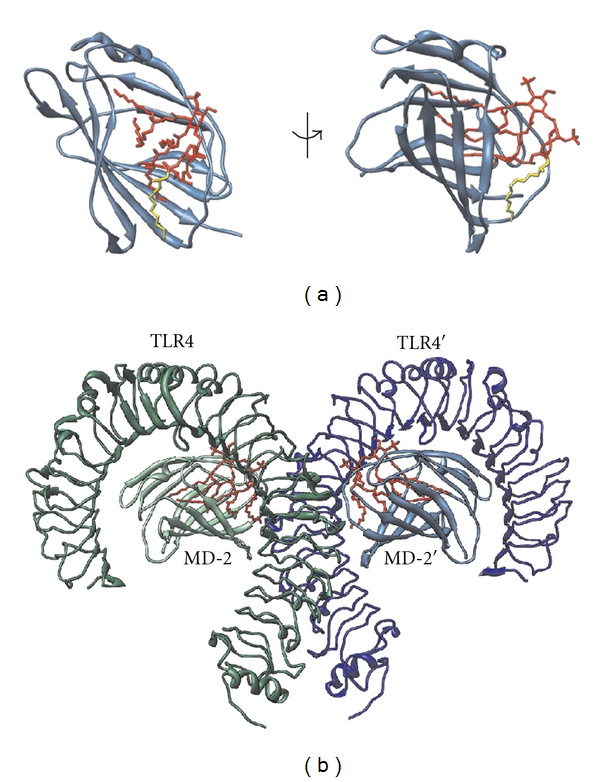
TLR4/MD-2 receptor complex recognizes and binds endotoxin. (a) MD-2 (shown in blue ribbons) is a soluble protein with a large hydrophobic pocket that directly binds bacterial endotoxin (red). One of the acyl chains of endotoxin (yellow) remains outside the hydrophobic pocket and mediates crucial interactions with TLR4 that bind the TLR4/MD-2 heterodimer together. Left: direct view of the MD-2 hydrophobic pocket. Right: side view showing the protruding endotoxin acyl chain. (b) The TLR4/MD-2/endotoxin heterodimer. Only the extracellular domains of TLR4 whose crystal structures were determined are shown [16].

TLRs are homologues of Toll, a receptor found in insects, that is involved in establishing dorsoventral polarity during embryogenesis as well as in immune response against fungal infections [11, 12]. The first discovered human Toll homologue was TLR4. It recognizes endotoxin (i.e., lipopolysaccharide), an outer membrane component of Gram-negative bacteria, that is composed of a conserved amphipathic lipid A component and of variable polysaccharides. The mechanism of TLR4 activation is quite complex and (unlike other TLRs) involves several auxiliary proteins (LBP, CD14) as well as a coreceptor (MD-2) [3] (Figure 1). It is in fact MD-2 and not TLR4 that directly recognizes and binds endotoxin [13, 14]. MD-2 is a soluble protein with a large hydrophobic pocket that represents the binding site for the acyl chains of lipid A. Lipid A is usually composed of 6 acyl chains, but only 5 of them bind into the hydrophobic pocket of MD-2. The 6th acyl chain protrudes out of the pocket and interacts with hydrophobic residues on TLR4. These interactions are crucial for MD-2/TLR4 heterodimerization and therefore prerequisite for the activation of the TLR4 signaling cascade [15, 16]. The endotoxin/MD-2/TLR4 heterodimer can, unlike other TLR signaling complexes, recruit two distinct intracellular adaptor proteins (i.e., MyD88/TIRAP and TRIF/TRAM) and can therefore activate two parallel signaling pathways and trigger the transcription of both proinflammatory cytokines as well as type I interferons [3]. Immune effects of TLR4 activation are indeed extensive; LPS alone can activate over 1000 genes [17]. It is therefore not too surprising that TLR4 activation affects not only the immune response against invading Gram-negative bacteria but is also involved in chronic inflammation, autoimmune diseases, and malignancies. TLR4 signaling in cancer is considered a double-edged sword. If TLR4 is activated on immune cells, it can enhance anti-tumor immunity. On the other hand, chronic inflammation is a major risk factor in cancer development [18].

## 3. TLR4 Expression in Cancer Cells

Progress in cancer research over the past decade has been immense, and the original fundamental characteristics of cancer (sustained proliferative signaling, evasion of growth suppressors, resistance to cell death, replicative immortality, induction of angiogenesis, invasion, and metastasis) [19] have recently been revisited and updated. Evasion of immune destruction rises as a new emerging hallmark of cancer [20]. Tumors utilize multiple mechanisms that help them turn the immune balance in their favor. They can secrete immunosuppressive cytokines (TGF*β*, IL-10, etc.), express antiapoptotic molecules, or downregulate tumor antigens and MHC1 expression [1]. TLRs are expressed by a variety of tumor cell lines, both in mouse and in human (Tables 1 and 2). Many of them are not limited to a single TLR but rather utilize an assortment of different TLRs (similarly to immune cells). 

Expression of TLR4 was confirmed by RT-PCR and FACS analysis on a large number of murine tumor cells, such as colon, breast, prostate, lung, and melanoma cancer cells. TLR4 signaling was shown to be unimpaired and could induce the synthesis of soluble immune mediators that could help the tumor to withstand the immune attack [8]. MC26 cells, for example, were shown to express functional TLR4 that (when activated by endotoxin) triggered activation of NF-*κ*B, ERK, and JNK kinases as well as the synthesis of iNOS, IL-6, and IL-12p70 [8]. iNOS and IL-6 have immunosuppressive effects [32–34], but IL-12p70 is generally not considered favorable for tumor development since it activates NK cells, induces T-cell proliferation, and promotes specific allogenic CTL reactions [35]. Some papers indicate that IL-12p70 can also have suppressive effects on allogenic or tumor-specific CTL generation [36, 37], but since evidence undisputedly demonstrates anti-tumor effects for IL-12 its production by tumor cells is possibly just a side product of TLR4 activation and subsequent NF-*κ*B activation. 

Supernatants from endotoxin-stimulated tumor cells were shown to inhibit T-cell proliferation and NK-cell cytotoxicity. Furthermore, blockade of tumor TLR4 signaling with anti-TLR4 siRNA or with inhibitory TLR4 peptide treatment prolongs the survival of MC26-bearing mice [8]. 

Functional TLR4 signaling was also demonstrated on human tumor cells. On colon carcinoma cells TLR4 signaling, in addition to production of immunosuppressive factors, also improved tumor cell apoptosis resistance [24]. Moreover, endotoxin stimulation of human prostate epithelial cancer cells elicited production of immunosuppressive and proangiogenic factors (TGF-beta and VEGF, resp.) [38].

TLR4 is expressed not only on malignant cells but also on normal tissues and benign tumors [30, 31]. Much remains to be studied concerning the function of TLR4 on normal nonimmune tissues in correlation with cancer development. But we must not forget to examine the expression of other contributing proteins in the TLR4 signaling cascade, for example, the adapter protein MyD88 (myeloid differentiation 88) that is essential for pro-inflammatory signaling. Although TLR4 expression was shown in normal ovarian epithelium, MyD88 was not expressed, therefore rendering TLR4 signaling via the proinflammatory MyD88-dependent pathway nonfunctional [9, 30]. Similar observation was made in a variety of colorectal carcinoma cell lines where tumor cells expressed TLR4 but failed to coexpress CD14, an important auxiliary protein in the endotoxin receptor complex [39] (Table 3). 

## 4. Chronic Inflammation Mediated by TLR4 in Cancer Development and Progression

Numerous links exist between inflammation and tumor development [18]. At the same time inflammatory cytokines are indispensable for immune cell activation and antitumor function. Therefore, there is an apparent contradiction when we consider the role of inflammation in cancer. It is plausible that part of the answer to this puzzle lies not in the inflammatory stimulation *per se* but in its timing, duration, and intensity. 

Chronic inflammation is often associated with cancer and can be the result of different causes, such as autoimmune disease or microbial infection. 

An example of microbial infection that can predispose an individual to cancer development is *Helicobacter pylori* infection. Infection with *H. pylori* is a known risk factor in gastric cancer and has been classified as a human carcinogen by the International Agency for Research on Cancer [45]. *H. pylori* infection is chronic and persistent, because *H. pylori* has the ability to evade immune system recognition. It has unusual endotoxin that exhibits very low endotoxic activity compared to the more common hexa-acylated form of endotoxin, usually found in enterobacteria (e.g., *Escherichia coli*) [46]. In spite of the inability to stimulate TLR4 on its own, *H. pylori* actively promotes inflammation by upregulating TLR4 expression via TLR2 and MEK1/2-ERK1/2 pathway giving way to TLR4 activation by endotoxin from other bacteria that pass through the gastrointestinal tract [47, 48]. TLR4 expression was indeed observed on gastric carcinoma tumor cells as well as on gastric epithelium with intestinal metaplasia and dysplasia [42]. 

Persistent inflammation is also a characteristic of colitis-associated neoplasms. Patients with ulcerative colitis have a five to eight times higher risk of developing colorectal cancer than the rest of the population [49, 50]. TLR4 expression is upregulated in colitis-associated cancer lesions from patients with ulcerative colitis but not in the surrounding tissue [51]. TLR4 seems to promote the development of colitis-associated colorectal tumors, and mice deficient in TLR4 are markedly protected against the development of neoplasia [52]. The reason behind this phenomenon could lie in the TLR4-Cox2-PGE_2_ signaling axis. Cyclooxygenase-2 (Cox-2) is aberrantly expressed in the majority of colorectal tumors and is (along with its enzymatic product prostaglandin E_2_) involved in the development of colorectal cancer [53]. It was recently shown that oral administration of high dosages of PGE_2_ can by-pass the protective effect exhibited by TLR4-deficient mice, which implicates PGE_2_ as an important TLR4 downstream molecule in colorectal cancer development as well as a potential target for more effective prevention of colitis-associated colorectal cancer [54].

TLR4 also has the potential to become a disease progression marker in patients with colon cancer or premalignant lesions [55] as well as a biomarker of the aggressive tumor phenotype in laryngeal carcinoma and breast cancer [41, 56]. Its high expression correlates with poor prognosis in colorectal cancer patients [57] and in murine models [58]. Furthermore, TLR4 is associated with liver metastasis; researchers showed an increase in TLR4 expression in steatotic murine livers following diet-induced obesity. In a metastatic model of colorectal cancer animals with steatotic livers had increased metastatic tumor mass within the liver compared to lean controls. Silencing of TLR4 on tumors lowered the tumor burden, indicating that tumor cell TLR4 signaling promotes metastatic growth [58]. On the contrary other studies concerning colorectal carcinoma showed correlation between reduced TLR4 expression and increased metastatic potential of the tumor [39].

TLR4 is associated with metastasis also in other types of cancer, such as melanoma, where TLR4 activation induces cell migration [28], and prostate cancer. It was shown that highly metastatic human prostate cancer cell lines, such as PC3 or DU145, express higher levels of TLR4 compared to poorly metastatic cell lines. Moreover, downregulation of TLR4 expression by siRNA can inhibit prostate cancer cell invasion *in vitro* and can improve survival of tumor-bearing animals [59]. Similar results were shown in human breast cancer cell line, where downregulation of TLR4 significantly reduced tumor cell proliferation [23]. 

Conversely, another study [60] reports a decrease in TLR4 expression in human prostate tissue samples that correlates with histopathological grade of prostate cancer. TLR4 expressed in normal and low-grade tumors could therefore be a contributing factor in chronic inflammation that promotes carcinogenesis [61], while decreased TLR4 expression in more aggressive high-grade tumors could result from loss of cell differentiation that accompanies cancer progression [60].

A similar phenomenon, though with a different underlying cause, can be seen in the case of cervical cancer, where Yu and coworkers [62] observed downregulation of TLR4 expression during progression of cervical neoplasia. They have attributed this downregulation to the immunosuppressive effect that persistent human papilloma virusinfection has on the host immune response [62]. A degree of prudence is therefore recommended when conclusions are made from the data currently available, because of major discrepancies between studies with respect to different species, cell culture, or cancer type studied.

## 5. Endogenous TLR4 Ligands Responsible for TLR4 Signalization in Cancer 

But what activates TLR4 signaling—is it bacterial endotoxin or perhaps other ligands? Endotoxin is ubiquitously present in air, gut, and epithelial surfaces, and perioperative exposure to it is associated with accelerated metastatic tumor growth [63]. Metastases could be the consequence of activation of the TLR4 signaling pathway that results in reduced apoptosis and increased proliferation of metastatic tumor cells. Killeen and coworkers [64] recently studied the role of endotoxin and TLR4 in invasion of extracellular matrix (ECM) and have shown that endotoxin promotes tumor cell ECM adhesion and invasion through activation of the urokinase plasminogen activator system (a serine protease that turns plasminogen into enzymically active plasmin responsible for blood clot degradation).

It is undisputed that the presence or absence of TLR4 expression on tumor (as well as nontumor) cells can influence different stages of carcinogenesis. Although many reports show clear correlation between chronic microbial infection and cancer initiation (e.g., *H. pylori* infection), others fail to provide evidence of the presence of endotoxin or other TLR4 ligands at cancer initiation sites. An important role is therefore attributed to different molecules of host origin that have lately arisen as potential endogenous ligands of TLR4. These proposed endogenous molecules include different components of the extracellular matrix, intracellular proteins, or modified lipids or lipoproteins (summarized in Table 4). Interestingly, many of them are proposed to activate both TLR4 and TLR2 without having any substantial structural similarity to their natural ligands (endotoxin or lipopeptides, resp.). 

Because many (if not most) of the studies describing putative endogenous TLR4 ligands (Table 4) used recombinant proteins and/or commercial reagents with undetermined levels of residual endotoxin, it is reasonable to raise concerns about the purity of the putative ligands used in experiments. The most common methods used to exclude potential endotoxin contamination are the limulus amebocyte lysate (LAL) test and endotoxin neutralization with polymyxin B (PMB). Some researchers demonstrate that their proposed TLR ligands lose their activating capacity after exposure to elevated temperatures. But as described in an excellent review by Erridge [104], these methods have a major shortfall when used in studies describing novel endogenous TLR4 ligands. LAL test, for example, is unable to detect endotoxin in the presence of endotoxin-binding molecules. Furthermore, molecules that bind endotoxin can also prevent its inactivation by PMB. As for the heat sensitivity, the biological activity of endotoxin can be greatly reduced by elevated temperatures. 

High-mobility group box-1 protein (HMGB1) is a putative TLR4 ligand implicated in cancer. HMGB1 is a nuclear DNA-binding protein that is actively secreted from cells following cytokine stimulation or passively released during cell death. It signals through the receptor for advanced glycation end products (RAGE) [105] and has been implicated in a variety of immune processes and pathological conditions including cancer [106, 107]. In the past few years many studies reported signalization of HMGB1 through TLR4 and declared HMGB1 an endogenous ligand of TLR4 [79, 80, 107]. HMGB1 is connected in several ways to tumor progression and metastasis [105]. On the other hand, HMGB1 released from irradiated or doxorubicin-/oxaliplatin- treated cells can improve immunogenicity of dying tumor cells and therefore help improve tumor antigen presentation [107]. A substantial number of studies show that HMGB1 however binds agonists of TLR, predominantly anionic molecules such as LPS [108], poly(IC), and CpG ODN that activate TLR4, TLR3, and TLR9; therefore, it may act as a chaperone [109, 110], similar to CD14, which stimulates activation of TLR4, TLR3, TLR7, and TLR9 by their agonists [111–113]. Additionally HMGB1 produced in mammalian cell cultures and therefore devoid of bacterial contaminants or endogenous danger signals does not activate TLR4 (unpublished observation). It should therefore be reconsidered whether these TLR4 ligands are not in fact just endotoxin-binding or endotoxin-sensitizing molecules without the intrinsic capability of binding and activating TLR4 on their own [104]. 

It is difficult to comprehend the multitude of the proposed TLR4 agonists that bear no structural similarity to the lipid A moiety of the LPS that is the only TLR4 agonist that has been prepared by chemical synthesis and whose molecular mechanism of activation is known [15, 16]. With respect to the plausible molecular mechanism of the direct activation of TLR4/MD-2 signaling complex oxidatively modified endogenous lipids seem to be the most likely ubiquitous endogenous agonists (Manček-Keber, manuscript in preparation).

## 6. Breaking the Immune Tolerance of Tumors by TLR4 Stimulation

Toll-like receptor activation is the trigger that sets the immune system into action. The application of TLR ligands in cancer therapy is therefore an attractive possibility that has been intensively studied in the past years in the context of cancer treatment or prevention (as anti-tumor vaccine adjuvants). Macrophages stimulated by endotoxin respond by secretion of chemokines and proinflammatory cytokines, including TNF*α* and interleukin-1*β*, which coordinate local and systemic inflammatory responses [17]. Dendritic cells, stimulated by endotoxin, secrete IL-12, which is important in anti-tumor immunity [114]. Furthermore, TLR4 stimulation induces DC maturation and antigen presentation, which has important effect on adaptive immune responses [4]. TLR stimulation influences antigen processing and presentation [115] by affecting the expression of costimulatory molecules on the surface of antigen-presenting cells as well as by controlling antigen uptake [116, 117] and phagosome maturation [118]. In addition to presenting antigens to lymphocytes, mature DCs are also capable of activating cancer-specific natural killer and NKT cells [119]. Inversely, TLR-stimulated NK cells facilitate in immature DC activation and maturation [120] and help intensify DC-mediated antitumor immune responses [121]. 

Tumors consist in large part not only of tumor but also of immune cells. It is therefore reasonable to assume that direct application of TLR ligands will affect both types of cells. TLR stimulation will possibly have even greater effect on the immune cell population, since not all tumor cells express TLR or the expression varies depending on the developmental stage of the tumor.

This is evident form an example of Bacillus Calmette-Guerin (BCG), an attenuated strain of *Mycobacterium bovis* that is used in the current treatment of nonmuscle invasive bladder cancer [122]. BCG promotes dendritic cell maturation, and this effect is TLR4 (as well as TLR2) dependent [123]. Furthermore, BCG can induce expression of TNF-related apoptosis-inducing ligand (TRAIL) on tumor infiltrating dendritic cells, therefore rendering them cytotoxic against tumor cells [124]. 

Another example of an immune activator of microbial origin that promotes dendritic cell maturation is the streptococcal agent OK-432. OK-432 is a preparation of a killed low-virulence strain of *Streptococcus pyogenes* that has been successfully used for over 30 years as an immunotherapeutic agent in different malignancies [125]. Its mechanism of action apparently involves TLR4 activation, since OKA-432 does not inhibit tumor growth on TLR4 knockouts as it does on wild-type mice. Moreover, patients with head and neck cancer responded to OK-432 treatment combined with fluoropyrimidine chemotherapy and radiation significantly better if they expressed TLR4 and MD-2 mRNA (compared to patients without TLR4 or MD-2 expression) [126, 127].

Stimulation of TLR4 on tumor cells can give contradicting results in terms of cancer progression versus treatment. The outcome seems to be species, tissue, and tumor type dependent. While TLR4 stimulation is on one hand associated with cancer progression (discussed above), it can also lead to anti-tumor immune response. B16 melanoma cells, for instance, that were stimulated with endotoxin *in vitro* exhibit reduced capability of inducing tumor growth *in vivo*. This response was totally independent of TLR4 expression by nontumor cells. *In vitro* stimulated tumor cells seem to differentially influence the phenotype of tumor infiltrating lymphocytes (TILs) so that TILs produced elevated levels of IFN-gamma and reduced levels of IL-10, thus favorably affecting the intratumoral cytokine balance [10].

## 7. Radio- and Chemotherapy Can Enhance Antitumor Immunity by Providing TLR Ligands

Combining immunotherapy and radiation is a new, compelling approach to cancer therapy. Though radiation is considered mostly immunosuppressive, it is noted also for its immunostimulatory effects. Patients therefore benefit from radiation therapy not only because it directly damages tumor cells but also because suppressor T-cell populations appear to be more radiosensitive than effector T lymphocytes [128]. Radiation can benefit anti-tumor immunity also by increasing expression of inflammatory cytokines by dendritic cells, therefore affecting their phenotype and function [129]. Dendritic cells are critical for anti-tumor immunity because of their ability to cross-present tumor antigens to specific CD8+ T lymphocytes. For efficient antigen cross-presentation, DCs need to receive appropriate stimulation through innate immune receptors. Since immature DCs can induce anti-tumor immunity when administered into irradiated tumors without the addition of TLR ligands [130], radiation was hypothesized to provide the necessary stimulus. 

**Figure 2:**
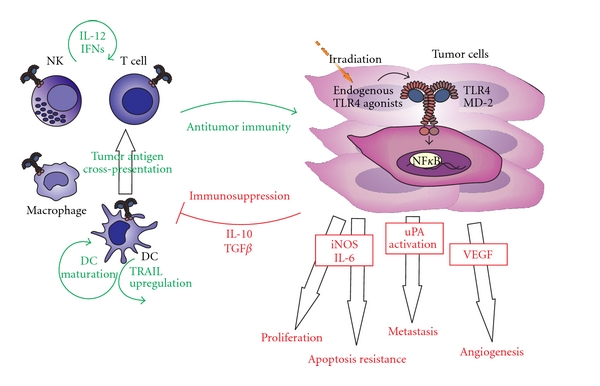
TLR4 signaling in cancer—a struggle of antitumor immunity against cancer proliferation and immune evasion. TLR4 signaling on immune cells can enhance anti-tumor immunity by different mechanisms, including IL-12 or IFN*γ* upregulation and promotion of DC maturation and function (left side of the figure, depicted in green). On the other hand, TLR4 signaling on tumor cells can increase their tumorigenic potential (right side of the figure, depicted in red).

Apetoh et al. [107] recently proposed that HMGB1, which is released from irradiated tumor cells, acts as an endogenous TLR4 ligand. They demonstrated that TLR4 is essential for efficient tumor antigen cross-presentation following radio- or chemotherapy and proposed that HMGB1 binds and activates TLR4 on DCs. HMGB1 could therefore activate DCs and prevent the accelerated degradation of the phagocytosed tumor antigens within DCs promoting efficient tumor antigen processing and cross-presentation [107] (Figure 2). 

The crucial role of TLR4 in immunostimulatory effects of radiation was also emphasized in a study by Paulos et al. [131], where they demonstrated elevated serum levels of endotoxin in mice following whole body irradiation. They showed that microbial endotoxin that translocated from the radiation-injured gut was responsible for enhanced anti-tumor effect of radiation. Moreover, radiation had diminished effect on tumors following removal of translocated endotoxin or in mice that were defective in the TLR4 signaling pathway [131]. These findings could be especially relevant for the treatment of gastrointestinal malignancies.

## 8. Cancer Vaccines Utilizing TLR4 Activation

Tumor cell lysates or purified tumor-associated antigens for vaccines have been used for therapeutic or prophylactic cancer vaccine. Although cell lysates contain endogenous danger signals that act as adjuvants, strong response against tumor-associated antigens requires additional stimulation of adaptive immune response by Toll-like receptor agonists. Agonists of TLR9 (CpG ODN), TLR3 (poly(IC), and TLR4 (endotoxin analogues) have been used to increase the innate immune response and activate antigen-presenting cells of the host. TLR4 is particularly important for development of a strong adaptive immune response by stimulation of the antibody class switching, affinity maturation, and formation of memory cells [132]. TLR4 is expressed on follicular dendritic cells that are essential for the affinity maturation in germinal centers [133, 134]. Systemic effect and toxicity of LPS preclude its application for cancer immunotherapy that started by the early attempts by William Coley. MPLA is a monophosphorylated lipid A derivative that has several orders of magnitude lower toxicity than lipid A and was reported to preferentially activate TRIF-dependent pathway [135]. MPLA has been registered as a vaccine adjuvant and used in clinical vaccines, such as Cervarix against human papilomavirus. MPLA is the only TLR4 agonist that has been clinically tested as an adjuvant for cancer vaccines. Results in clinical trials have been modest but seem to be much better if the vaccines are used in early stages of the disease, such as, for example, therapy of non-small-cell lung carcinoma (NSCLC) using MAGE-3 antigen combined with MPLA-based adjuvant AS02B rather than in late stages, when the immune system of patients is already severely compromised (reviewed in [136]). Additional alternative therapeutic approaches are based on combination of TLR4 agonist as a vaccine adjuvant with tumor-associated antigens in combination with radio- or chemotherapy or autologous dendritic cell therapy.

## 9. Conclusions

TLR signaling triggers immune cell activation and maturation and is indispensable for the efficient immune response against the pathogenic microorganisms as well as against malignant cells. An effective immune system is most important in the early stages of carcinogenesis when cancerous cells are few and are not limited to less immunogenic cell clones. If immunosurveillance against malignantly transformed cells is unsuccessful in the early stage, tumors quickly outgrow the immune cell cytotoxic capabilities. TLR4 expression by tumor cells can be a contributing factor that promotes tumor cell proliferation, survival, or immunosuppression. 

Therapeutic interventions at the level of TLR4 stimulation is a double-edged sword since different studies demonstrate positive as well as negative effects of TLR4 stimulation on cancer development or treatment. Harnessing the beneficial effects of TLR4 stimulation while eliminating the negative ones remains the challenge for cancer researchers.

## Figures and Tables

**Table 1:** Murine tumor cell lines that express TLR4.

Tumor type	Murine tumor cell line	References
Breast cancer	4T1	[8]
Colon cancer	MC26	[8]
Glioma	GL261	[21]
Lung cancer	LLC1	[8]
Melanoma	B16	[8]
Prostate cancer	RM1	[8]

**Table 2:** Human tumor cell lines that express TLR4.

Tumor type	Human tumor cell line	References
Bladder cancer	T24	[22]
Breast cancer	MDA-MB-231	[23]
Colon cancer	SW480, HT29, KM20	[24, 25]
Laryngeal and oral cancer	PCI-1, PCI-30	[26]
Melanoma	SkMEL-28, BN1, 9923M, ME5, ME16, ME17	[27, 28]
Neuroblastoma	NB-1	[29]
Ovarian cancer	SKOV3, AD10, A2780, CP70	[9, 30, 31]

**Table 3:** Human tumors expressing TLR4.

Tumor type	References
Adrenocortical cancer	[40]
Breast cancer	[41]
Bladder cancer	[22]
Colon cancer	[24, 25, 39]
Gastric cancer	[42]
Laryngeal cancer	[26]
Lung cancer	[43]
Melanoma	[27, 28]
Neuroblastoma	[29]
Ovarian cancer	[9, 30, 31]
Prostate cancer	[44]

**Table 4:** 

Proposed endogenous TLR4 ligand		Reference
Advanced glycation end product low-density lipoprotein	AGE-LDL	[65]
Angiotensin II		[66, 67]
Beta defensin		[68, 69]
Biglycan		[70, 71]
Calprotectin		[72]
Ceramide		[73]
Fibrinogen		[74, 75]
Fibronectin extra domain A	F-EDA	[76, 77]
High-mobility group box 1	HMGB1	[78–81]
Heat shock protein	HSP	[82–85]
Heparan sulfate		[86]
Hyaluronan		[87–92]
Minimally modified (oxidized) low-density lipoprotein	mmLDL	[93–95]
Myeloid-related protein-8/14	MRP-8/14	[96]
Oxidized Palmitoyl-arachidonoyl-phosphatidylcholine	OxPAPC	[97, 98]
Pancreatic adenocarcinoma upregulated factor	PAUF	[99]
Serum amyloid A		[100]
Saturated fatty acid	SFA	[101]
Surfactant protein A		[102]
Tenascin-C		[103]
